# Hemophagocytic Lymphohistiocytosis as the Initial Presentation of Acute HIV Infection: A Case Report

**DOI:** 10.7759/cureus.114263

**Published:** 2026-08-10

**Authors:** Siham Karrati, Amina Bendriouich, Salma Rouhi, Wafa Quiddi, Sanae Sayagh

**Affiliations:** 1 Hematology Laboratory, Mohammed VI University Hospital, Cadi Ayyad University, Marrakech, MAR; 2 Research Center for Childhood, Health and Sustainable Development, Cadi Ayyad University, Marrakech, MAR

**Keywords:** acute hiv infection, cytokine storm, hemophagocytic lymphohistiocytosis, hemophagocytosis, hiv

## Abstract

Hemophagocytic lymphohistiocytosis (HLH) is a rare but severe disorder of immune dysregulation associated with overwhelming systemic inflammation and multiorgan dysfunction. Infectious diseases are among the most common triggers of secondary HLH, with viral infections predominating. HLH is a recognized complication of advanced HIV infection; however, it remains exceptionally rare during acute HIV infection. We report the case of a previously healthy 19-year-old man admitted with a two-week history of fever, asthenia, myalgia, anorexia, and significant weight loss. Physical examination revealed pallor and cervical lymphadenopathy. Laboratory investigations showed pancytopenia, marked hyperferritinemia, hypertriglyceridemia, elevated liver enzymes, and an increased lactate dehydrogenase level. Bone marrow examination demonstrated hemophagocytosis. The overall findings were consistent with HLH, as five of the eight HLH-2004 diagnostic criteria were met. An extensive workup for secondary HLH triggers, including infectious, autoimmune, and malignant causes, identified acute HIV-1 infection with high-level viremia and CD4 T-cell lymphopenia. The diagnosis of HLH secondary to acute HIV-1 infection was established, and the patient was promptly started on antiretroviral therapy and corticosteroids, resulting in favorable clinical and laboratory outcomes. Although rare, HLH may present as the initial manifestation of acute HIV infection. Early recognition of this association is crucial, as prompt diagnosis and treatment of the underlying HIV infection can significantly improve outcomes.

## Introduction

Hemophagocytic lymphohistiocytosis (HLH) is a severe disorder resulting from immune dysregulation, in which persistent activation of cytotoxic lymphocytes and macrophages triggers an uncontrolled cytokine-driven hyperinflammatory response that can rapidly lead to tissue damage and multiorgan failure [1,2]. The diagnosis of HLH remains challenging due to its nonspecific and heterogeneous clinical presentation, requiring a high index of clinical suspicion [2,3]. The diagnosis is based on the HLH-2004 diagnostic criteria, which require fulfillment of at least five of eight clinical, laboratory, and pathological criteria [1,3]. Early recognition and prompt initiation of appropriate therapy are essential to improve patient outcomes and reduce mortality [1,3,4]. Based on its underlying etiology, HLH is categorized as either primary (familial) or secondary (acquired). Primary HLH generally follows an autosomal recessive mode of inheritance and results from pathogenic variants in genes involved in cytotoxic lymphocyte function and immune homeostasis, typically presenting during childhood. In contrast, secondary HLH occurs more frequently in adults and arises from acquired immune dysregulation triggered by infections, malignancies, or autoimmune and autoinflammatory disorders [2,3].

Secondary HLH is most frequently associated with infectious triggers, particularly viral infections, with Epstein-Barr virus (EBV) being the most commonly implicated pathogen [2,4-6]. HLH associated with HIV infection is rare and usually occurs in advanced stages of the disease, often in the context of opportunistic infections or HIV-related malignancies [5,6]. Its occurrence during acute HIV infection is exceedingly uncommon and has been described in isolated cases to date [6-12]. This association is of particular clinical interest due to its diagnostic challenge and potential severity.

Here, we report a case of HLH presenting as the initial manifestation of acute HIV infection in a 19-year-old man, with favorable clinical and laboratory outcomes following treatment with antiretroviral therapy and corticosteroids. This case emphasizes the importance of early recognition of HLH in patients presenting with fever and cytopenias and highlights the need to consider acute HIV infection in the etiologic workup of HLH.

## Case presentation

A previously healthy 19-year-old man was admitted to the emergency department with a two-week history of fever, anorexia, asthenia, myalgia, and unintentional 7 kg weight loss. There were no respiratory, gastrointestinal, urinary, or other localizing symptoms to suggest a focal source of infection.

Physical examination at admission revealed an alert, febrile patient (39.5°C) with stable hemodynamic and respiratory parameters, including a blood pressure of 126/70 mmHg, a heart rate of 80 beats per minute, a respiratory rate of 17 breaths per minute, and an oxygen saturation of 97%. He had marked mucocutaneous pallor and cervical lymphadenopathy, without hepatomegaly or splenomegaly. The remainder of his examination was normal.

Initial complete blood count revealed pancytopenia, with a leukocyte count of 2,190 cells/mm³, normochromic normocytic non-regenerative anemia with a hemoglobin level of 7.9 g/dL, and a platelet count of 30,000 cells/mm³. Peripheral blood smear confirmed pancytopenia, with no circulating blasts or atypical cells. The coagulation profile was unremarkable, with normal prothrombin time, activated partial thromboplastin time, and fibrinogen levels. Biochemical analysis showed elevated C-reactive protein, marked hyperferritinemia, hypertriglyceridemia, elevated liver enzymes (aspartate aminotransferase and alanine aminotransferase), elevated lactate dehydrogenase, and hypoalbuminemia.​​​​​​ Renal function was preserved. Blood cultures were negative.

**Figure 1:**
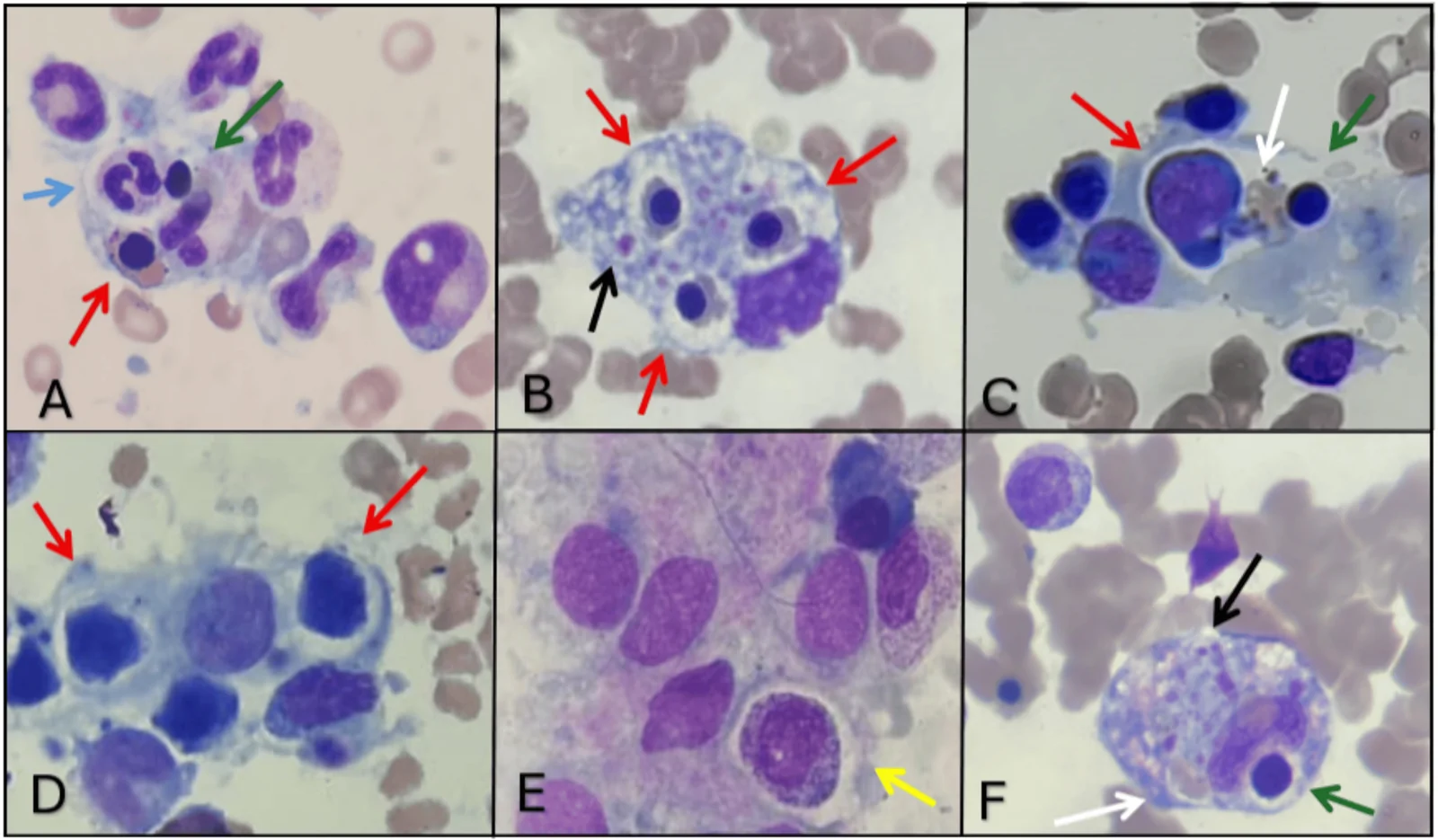
Bone marrow aspirate examination (May–Grünwald–Giemsa stain, 1,000× magnification). (A) Hemophagocytic histiocyte engulfing a neutrophil (blue arrow), a lymphocyte (green arrow), and an acidophilic erythroblast (red arrow). (B) Hemophagocytic histiocyte engulfing platelets (black arrow) and acidophilic erythroblasts (red arrows). (C) Hemophagocytic histiocyte containing a proerythroblast (red arrow), an erythrocyte (white arrow), and a lymphocyte (green arrow). (D) Hemophagocytosis of erythroblasts (red arrows). (E) Hemophagocytosis of a myelocyte (yellow arrow). (F) Histiocyte phagocytosing platelets (black arrow), an erythrocyte (white arrow), and a lymphocyte (green arrow). These findings illustrate hemophagocytosis, an important pathological finding that supports the diagnosis of hemophagocytic lymphohistiocytosis in conjunction with compatible clinical and laboratory findings.

Given the strong clinical and laboratory suspicion of HLH, a bone marrow aspirate was performed, revealing a normocellular marrow with numerous activated histiocytes exhibiting hemophagocytosis (Figure 1), with no evidence of malignancy, parasitic infection, or fungal elements.

Based on the patient’s clinical, laboratory, and bone marrow findings, the HScore was calculated at 281, corresponding to an estimated probability of >99% of reactive hemophagocytic lymphohistiocytosis. The diagnosis of HLH was established according to the HLH-2004 diagnostic criteria, with five criteria fulfilled.

An etiological workup was performed. Serologic testing for EBVs, cytomegalovirus, hepatitis B virus, hepatitis C virus, *Brucella* spp., and *Treponema pallidum* was negative. Polymerase chain reaction testing for *Leishmania* was negative. Tuberculosis was ruled out using a GeneXpert assay on respiratory samples. Autoimmune screening was negative for antinuclear and antineutrophil cytoplasmic antibodies. A contrast-enhanced cervicothoracoabdominopelvic CT scan was performed, demonstrating cervical lymphadenopathy with preserved benign morphological features. The affected lymph nodes were oval, well-defined, and showed homogeneous contrast enhancement with preserved central fatty hila. There was no evidence of central necrosis, calcifications, or adjacent cervical fat stranding. No mediastinal, abdominal, or pelvic lymphadenopathy or other imaging findings suggestive of malignancy were identified. HIV testing with a fourth-generation HIV Ag/Ab assay (Alinity s, Abbott) was positive, while the Western blot showed no detectable HIV antibody bands. Plasma HIV-1 RNA quantification revealed a viral load of 316,000 copies/mL, with a CD4+ T-cell count of 120 cells/mm³, confirming acute HIV-1 infection. After exclusion of alternative infectious, malignant, and autoimmune causes, acute HIV infection was considered the most likely trigger of HLH. The results of all laboratory investigations are summarized in Table 1.​​​​

**Table 1:** Summary of laboratory findings. EBV: Epstein–Barr virus; VCA: viral capsid antigen; IgM: immunoglobulin M; IgG: immunoglobulin G; EBNA: Epstein–Barr nuclear antigen; CMV: cytomegalovirus; HBsAg: hepatitis B surface antigen; anti-HBs: hepatitis B surface antibody; anti-HBc: hepatitis B core antibody; anti-HCV: hepatitis C virus antibody; HIV Ag/Ab assay: human immunodeficiency virus antigen/antibody assay; S/CO: signal-to-cutoff ratio; TPHA: *Treponema pallidum* hemagglutination assay; VDRL: Venereal Disease Research Laboratory test; SAT: standard agglutination test (Wright test); PCR: polymerase chain reaction; GeneXpert MTB/RIF: Xpert *Mycobacterium tuberculosis*/rifampicin assay

Parameter	Observed value	Reference range
Leukocyte count	2,190	4,000–10,000 cells/mm³
Hemoglobin	7.9	13–17 g/dL
Mean corpuscular volume	94	80–100 fL
Mean corpuscular hemoglobin concentration	35	32–36 g/dL
Reticulocyte count	11	20–80 × 10⁹/L
Platelet count	30,000	150,000–400,000 cells/mm³
C-reactive protein	82	<6 mg/L
Prothrombin activity	88	70–100%
Activated partial thromboplastin time	29.6	28–35 seconds
Fibrinogen	2.4	2–4 g/L
Ferritin	>33,511	30–400 ng/mL
Lactate dehydrogenase	3537	125–220 U/L
Triglycerides	610	<150 mg/dL
Aspartate aminotransferase	128	<35 U/L
Alanine aminotransferase	184	<50 U/L
Albumin	27	32–45 g/L
Urea	36	25–48 mg/dL
Creatinine	0.9	0.7–1.2 mg/dL
Blood cultures	Negative	Negative
EBV VCA IgM	0	S/CO <0.5
EBV VCA IgG	0	S/CO <0.75
EBV EBNA IgG	0	S/CO <0.5
CMV IgM	0	S/CO <0.85
CMV IgG	0	S/CO <6
HBsAg	Negative (0.32)	S/CO <1
Anti-HBs	0	S/CO <10
Total anti-HBc	Negative (0.21)	S/CO <1
Anti-HCV	Negative (0.57)	S/CO <1
TPHA	0	S/CO <1
VDRL	0	S/CO <1
SAT	Negative	<1:80
Leishmania spp. PCR	Negative	Negative
GeneXpert MTB/RIF	Negative	Negative
Antinuclear antibodies	Negative	Negative
Anti-neutrophil cytoplasmic antibodies	Negative	Negative
HIV Ag/Ab assay	Positive (1,203)	S/CO <1
HIV Western blot	No detectable HIV antibody bands	-
Plasma HIV-1 RNA	316,000 copies/mL	Not detected
CD4+ T-cell count	120	500–1,500 cells/mm³

The patient was treated with a once-daily antiretroviral regimen consisting of tenofovir disoproxil fumarate, lamivudine, and dolutegravir, in combination with high-dose dexamethasone (10 mg/m²/day for two weeks, tapered thereafter), administered according to the dexamethasone dosing schedule of the HLH-94 protocol. He showed a favorable clinical and laboratory course, characterized by resolution of fever, improvement in his general condition, and progressive normalization of the complete blood count (white blood cells: 5,620 cells/mm³; hemoglobin: 13.1 g/dL; platelets: 172,000 cells/mm³), inflammatory markers (ferritin: 204 ng/mL; triglycerides: 100 mg/dL; C-reactive protein: 3 mg/L), and liver function tests (alanine aminotransferase: 26 U/L; aspartate aminotransferase: 22 U/L). The HIV viral load became undetectable, accompanied by an increase in the CD4 T-cell count to 950 cells/mm³. After eight months of follow-up, the patient remained asymptomatic with no evidence of HLH relapse.

## Discussion

HLH is a potentially fatal condition driven by aberrant and persistent activation of cytotoxic T lymphocytes and natural killer cells. This immune dysregulation results in sustained release of pro-inflammatory cytokines and macrophage activation, leading to multiorgan involvement [1,2,13].

HLH presents significant diagnostic and therapeutic challenges due to its heterogeneous etiologies and nonspecific clinical presentation. Its manifestations frequently overlap with those of severe infections, malignancies, and autoimmune disorders, which may delay diagnosis and appropriate management [2,3].

According to the HLH-2004 criteria, the diagnosis of HLH is based on a composite set of eight clinical, laboratory, and immunologic parameters, of which at least five must be present: fever >38.5°C; splenomegaly; cytopenias affecting ≥2 peripheral blood cell lineages (hemoglobin ≤9 g/dL, platelet count <100 × 10⁹/L, and neutrophils <1.0 × 10⁹/L); fasting triglycerides ≥3.0 mmol/L and/or fibrinogen ≤1.5 g/L; hemophagocytosis in bone marrow, spleen, or lymph nodes with no evidence of malignancy; impaired natural killer cell activity; ferritin ≥500 μg/L; and soluble CD25 ≥2,400 U/mL [1,2,14,15]. In the present case, our patient fulfilled five of these criteria, including fever, pancytopenia, hypertriglyceridemia, hyperferritinemia, and numerous hemophagocytic figures on bone marrow examination. Natural killer cell activity and soluble CD25 levels were not assessed because these specialized assays were unavailable at our institution. Although these parameters may provide additional diagnostic support, their absence did not significantly affect the diagnosis in this case, as the patient fulfilled five of the eight HLH-2004 diagnostic criteria.

The HScore is a validated score developed to estimate the probability of reactive HLH, particularly in adults. It incorporates nine weighted variables, including known immunosuppression, fever, organomegaly, cytopenias, ferritin, triglycerides, fibrinogen, transaminase levels, and hemophagocytosis on bone marrow examination. The probability of reactive HLH ranges from less than 1% for an HScore below 90 to greater than 99% for an HScore above 250 [16]. While the HLH-2004 criteria remain widely used for HLH diagnosis, the HScore provides an individualized probability estimate by integrating multiple clinical, laboratory, and cytological parameters and may serve as a complementary diagnostic tool, particularly in adults with suspected HLH. In our patient, the HScore was 281, corresponding to a greater than 99% probability of reactive HLH, further supporting the diagnosis.

In adults, HLH most frequently occurs as a secondary disorder triggered by underlying conditions, particularly infections, malignancies, or autoimmune and autoinflammatory diseases [3,13]. While EBV remains the most frequently implicated pathogen in infection-associated HLH, HIV-related HLH is rare and is most commonly reported in patients with advanced immunosuppression, often in association with opportunistic infections or underlying malignancies [5,6,9,17]. The occurrence of HLH during acute HIV infection is even more uncommon and has been sporadically reported in the literature [6-12,18,19]. In this case, HLH was the initial clinical manifestation leading to the diagnosis of acute HIV infection.

During acute HIV-1 infection, massive viral replication and rapid systemic dissemination trigger intense activation of innate and adaptive immune responses, including dendritic cells, macrophages, natural killer cells, and CD8⁺ T lymphocytes, resulting in markedly increased levels of cytokines, including type I interferons, interleukin-15, tumor necrosis factor alpha, interferon-gamma, IL-6, IL-8, IL-18, and IL-10 [18,20]. This exaggerated immune response may fail to resolve, giving rise to a cytokine storm that perpetuates macrophage activation and hemophagocytosis, ultimately resulting in the clinical and laboratory manifestations of HLH [21].

Although uncommon, acute HIV infection may act as the sole trigger of HLH after exclusion of other infectious, malignant, and autoimmune causes. In our patient, an extensive etiological workup failed to identify any alternative cause of HLH. Although the initial CD4+ T-cell count was markedly reduced, profound but transient CD4+ T-cell depletion has been described during acute HIV infection and may result from massive viral replication, preferential infection and depletion of activated CD4+ T cells, particularly memory CCR5-expressing subsets, together with immune-mediated apoptosis and redistribution of circulating CD4+ T cells to lymphoid tissues [22,23]. In our case, the overall immunovirological profile, including a reactive fourth-generation HIV Ag/Ab assay, the absence of HIV-specific bands on Western blot, a high plasma HIV-1 RNA level, and rapid immune reconstitution following initiation of antiretroviral therapy, with recovery of the CD4+ T-cell count to 950 cells/mm³, was consistent with acute HIV infection rather than established chronic infection. Acute HIV infection was therefore the most likely precipitating factor, and the HLH was presumed to reflect the profound immune activation accompanying the early phase of HIV infection.

The management of secondary HLH generally relies on two complementary approaches: treatment of the underlying trigger and control of the hyperinflammatory response. In the setting of HIV infection, treatment primarily focuses on early initiation of antiretroviral therapy to control viral replication and restore immune function. In addition, it is recommended to treat the hyperinflammatory state with corticosteroids, with or without intravenous immunoglobulins [2,15]. In the current case, early recognition of acute HIV infection as the underlying trigger allowed prompt initiation of appropriate therapy, which was associated with favorable clinical and laboratory outcomes.

Overall, this case adds to the limited body of literature on HLH occurring in the setting of acute HIV infection and underscores the importance of increasing clinical awareness of this rare but clinically significant association.

## Conclusions

HLH is a rare and complex hyperinflammatory syndrome with a wide range of etiologies. This case highlights that acute HIV infection, although uncommon, should be considered a potential trigger of HLH, particularly when no alternative cause is identified. Early identification of the underlying trigger and prompt initiation of appropriate treatment are essential to control the hyperinflammatory response and improve clinical outcomes. Reporting such rare presentations may enhance clinician awareness, facilitating earlier diagnosis. Nevertheless, given the inherent limitations of a single case report, further studies are needed to better define the optimal management and clinical outcomes of HLH associated with acute HIV infection.
